# Recent Advances in Portable Biosensors for Biomarker Detection in Body Fluids

**DOI:** 10.3390/bios10090127

**Published:** 2020-09-18

**Authors:** Brian Senf, Woon-Hong Yeo, Jong-Hoon Kim

**Affiliations:** 1 School of Engineering and Computer Science, Washington State University, Vancouver, WA 98686, USA; brian.senf@wsu.edu; 2 Human-Centric Interfaces and Engineering Program, Wallace H. Coulter Department of Biomedical Engineering, George W. Woodruff School of Mechanical Engineering, Georgia Institute of Technology, Atlanta, GA 30332, USA; whyeo@gatech.edu

**Keywords:** portable biosensor, biomarkers in body fluids, portability, point-of-care

## Abstract

A recent development in portable biosensors allows rapid, accurate, and on-site detection of biomarkers, which helps to prevent disease spread by the control of sources. Less invasive sample collection is necessary to use portable biosensors in remote environments for accurate on-site diagnostics and testing. For non- or minimally invasive sampling, easily accessible body fluids, such as saliva, sweat, blood, or urine, have been utilized. It is also imperative to find accurate biomarkers to provide better clinical intervention and treatment at the onset of disease. At the same time, these reliable biomarkers can be utilized to monitor the progress of the disease. In this review, we summarize the most recent development of portable biosensors to detect various biomarkers accurately. In addition, we discuss ongoing issues and limitations of the existing systems and methods. Lastly, we present the key requirements of portable biosensors and discuss ideas for functional enhancements.

## 1. Introduction

**Figure 1:**
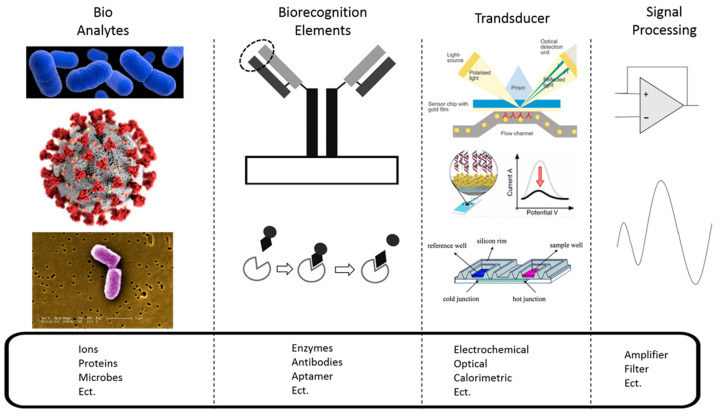
Overview of biomarker detection using biosensors. Bio-analytes from [3], Biorecognition elements from [4], Transducer images from [5,6,7].

Biosensors are a subset of chemical sensors, which transform chemical data into an analytical signal to monitor physiological and chemical analytes in the body. The chemical sensors are comprised of a chemical recognition system and a physicochemical transducer. Likewise, biosensors use a biochemical reaction as the recognition element [1] and convert a biological response into an electric signal [2]. The biosensor requires multiple components to measure these analytes and display information related to the analysis done. Figure 1 shows the schematic diagram of a biosensor composed of a biorecognition element, transducer, and signal processing unit. The biorecognition layer is the defining component that determines the specificity of the device. It’s also called the bio receptor, which is a molecular species that utilizes a biochemical mechanism for the recognition of analytes. The biorecognition element binds the analytes of interest to the sensor’s surface for the reaction. The reaction is then converted by the transduction mechanism. The transducer converts a biological signal to a measurable signal.

It converts one form of energy to another and can take many forms depending on design specifications. One common type of transduction mechanism used in biosensors is the electrochemical transducer. Many reactions with the biorecognition layer either produce or consume ions or electrons. This can cause changes in the electrical properties of a solution that can be measured and transduced to a signal processor. The signal processor is generally a computer or microprocessor which acquires the signal, then filters and amplifies the data. Electrical noise is inherent when processing data from the transducer. Thus, the signal processor typically subtracts the baseline noise from the transducer to amplify the signal of interest [2].

Fabrication of biosensors is a broad topic with many different strategies and techniques, depending not only on the analyte but also on the scale of the biosensor and the longevity intended. The first true biosensor was created in 1956 by Leland C. Clark Jr. The biosensor stemmed from an earlier invention on the oxygen electrode. To calibrate the electrode, he had been deoxygenating test solutions using the enzyme glucose oxidase and glucose, which was capable of removing the oxygen from the solution. He eventually realized that this procedure could instead be used to measure glucose concentration. This was done by immobilizing the glucose oxidase on the oxygen electrode. The concentration of air could then be directly mapped to the concentration of glucose in the solution [8]. Many biosensors follow a similar path now. The oxidation or reduction reaction produces a readable signal for the electrode to read as a current. Therefore, the current can be correlated with the concentration of the analyte of interest in the solution being tested. For enzyme biosensors, the enzyme must first be immobilized in a membrane which can then be coated onto an electrode. The electrode chosen is dependent on the desired reaction [9]. A detailed explanation of the wide range of fabrication for biosensors is outside of the scope of this work, but more information can be found in the “Handbook of Biosensor and Biosensor Kinetics” with an entire chapter dedicated to covering some of the recent techniques used for biosensor fabrication [10].

Technology advances have enabled biosensors to become more accurate, reliable, and sensitive to biomarkers and analytes. As these Biosensors become more advanced, there is also a movement to make them smaller and more portable. Portable devices make them available outside of the laboratory allowing for use in the field as well as transportation to third world countries where the inability for early, rapid detection of disease has a great negative effect not only on people’s lives but economies on the whole. Portable biosensors can also help prevent the spread of disease by mitigating the travel of diseased individuals. Currently, the diagnosis requires travel to a clinic as well as an extended wait period to receive the results. Subjects going in for tests can deal with white coat hypertension, which is a blood pressure difference of 20 mmHg systolic or 10 mm Hg diastolic between ambulatory and clinic blood pressures [11]. This goes to show that clinic visits can add a quantifiable amount of stress to a patient’s life and can even affect test results. Therefore, the demand for portable biosensors has increased, and research is focused on the development of small portable devices that would allow rapid, accurate, and on-site detection. However, the requirements of portability are not clearly defined, especially regarding the sample preparation methods. Its perspective needs to be reviewed in detail.

The use of portable biosensors in remote environments outside of laboratory and clinical settings often correlates with an individual who may not have the same level as training those in a clinical setting. Less invasive sample collection then becomes pertinent for true on-site diagnostics and testing. For non- or minimally invasive sampling, easily accessible body fluids such as saliva, sweat, blood, or urine, have been the sample of choice as they are among the easiest types of clinical samples to obtain. Blood has been the most common sample type as a part of a routine physical examination due to many disease-associated biomarkers and its less invasive collection procedure. Thus, it becomes a good source of biomarkers for portable biosensors. Recently, other body fluids, including saliva, urine, and sweat, have attracted much attention as samples for portable biosensors due to their abundant presence, diagnostic capabilities, and noninvasive sample extraction.

In this article, we review the most recent development of biomarkers for portable biosensor systems with various sample types, i.e., saliva, sputum, blood, breath, tears, urine, and sweat. In addition, some of the recent advances in the field of portable biosensor analyzed by sample type, as well as a few issues with the field are discussed. We present metrics on portability as for the biosensors and discuss ideas for improvement in portability classifications. The difference in bio-sensing strategies and analytical performance is discussed to provide a most recent overview of the portable biosensor development, with conclusions and future perspectives at the end.

## 2. Analytes

### 2.1. Saliva

**Figure 2:**
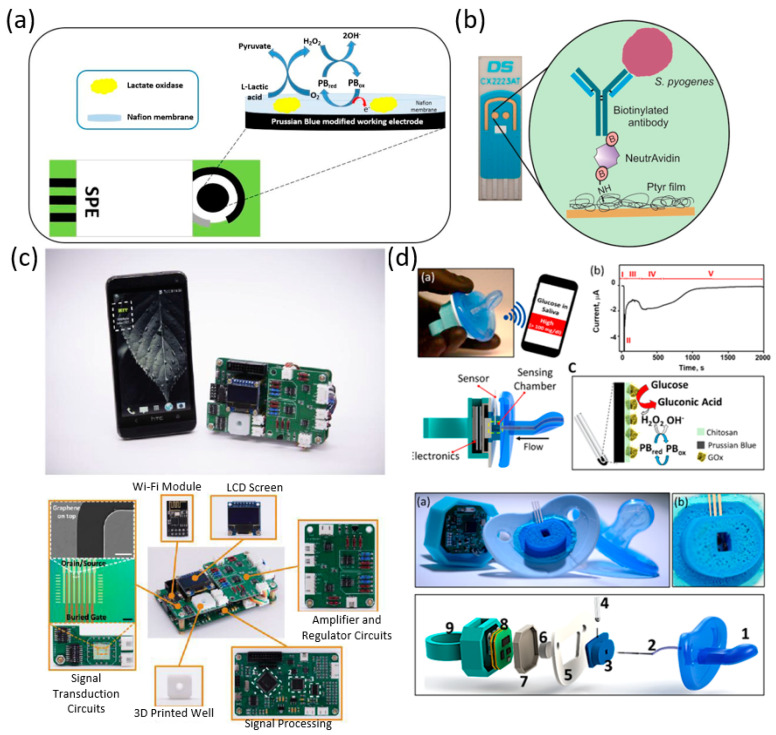
Portable biosensors for salivary diagnostics. (**a**) Schematic representation of SPE-PB-LOx Biosensor [14]. (**b**) Schematic of immunosensor against S. pyogenes [17]. (**c**) Images of aptameric GFET nanosensing system for cytokines detection [19]. (**d**) Glucose Pacifier sensing concept [25].

Recently, salivary diagnostics shows excellent promise as a convenient means for the early detection, prognosis, and monitoring post-therapy status, as saliva is 99% water and can be easily manipulated [12]. Screening biomarkers in the saliva is advantageous because the sample extraction is simple, noninvasive, stress-free, cost-effective, and precise. Additionally, since saliva sample collection does not require any special tools, it can even be done at home by patients themselves for continuous monitoring in necessity and easily integrated with a portable biosensor for point-of-care (POC) testing [13]. The use of saliva is broadening biosensor perspectives in disease diagnosis, clinical monitoring, and decision making for patient care. In this section, we summarize many types of salivary biomarkers for portable biosensors (Table 1). In addition, we describe the recent development of portable saliva biosensors along with their limits and ranges of detection (Figure 2).

Lactate is a useful biomarker that can be tested for in saliva. It can be used to diagnose metabolic disorders, monitor diabetes, and is a useful measurement for sports physiology. Petropoulos et al. proposed a screen-printed electrode modified with Prussian Blue. The sensor detects hydrogen peroxide produced by the reaction catalyzed by the lactate oxidase enzyme immobilized onto the electrode surface. Figure 2a presents the biosensor integrated with portable instrumentation showing a working range from 0.025–0.25 mM and a limit of detection (LOD) of 0.01 mM [14]. Roda et al. reported a 3D printed mini cartridge that can be used to turn a smartphone or tablet in a luminometer. The design allows for the detection of chemiluminescence derived from enzyme reactions. To prototype the sensor, they coupled lactate oxidase with horseradish peroxidase for the measurement of lactate in saliva and sweat. The biosensor reported a LOD of 0.5 mmol/L in saliva [15]. Yao et al. developed screen-printed carbon electrodes and electrochemical chambers on a hydrophilic cloth. A smartphone is then used to read the electrochemiluminescence signals. The biosensor reports a LOD of 0.35 mM and a dynamic range of 0.05–2.5 mM [16]. Streptococcus pyogenes is a bacterium which causes an estimated 700 million of infection per year. As there are no available vaccines yet, early detection of infection is crucial to prevent serious invasive infection, which has a mortality rate of 25%. Ahmed et al. reported a screen-printed gold electrode used to create a polytyramine-based immunosensor for the detection of Streptococcus pyogenes. Biotin tagged whole antibodies against Streptococcus pyogenes were conjugated to polytramine amine groups via biotin-NeutrAvidin coupling. They showed a working range of 100 to 10^5^ cells/10 µL for cumulative incubation and 100 to 10^4^ cells/10 µL single-shot incubation. An image of the proposed biosensor can be seen in Figure 2b [17]. Avian influenza viruses (AIV) are naturally occurring among birds and can be spread to humans, although infection in humans is rare. However, the possibility that the virus could change and begin spreading between people is of major concern making rapid detection and identification crucial for control of outbreaks [26]. Wang et al. demonstrated an AIV biosensor and its performance on infected poultry. The design was an impedance biosensor based on a combination of magnetic nanobeads coated with AIV subtype-specific antibody. The system is also integrated with a microfluidic chip interdigitated array microelectrode for transfer, detection, and measurement of the bio-nanobeads and virus complex in a buffer. The biosensor was reported to have a LOD of 1 × 10^2.2^ ELD_50_/mL where EDL_50_ is 50% egg lethal dose [18]. Elevated levels of cytokine biomarkers in bodily fluids have been reported in patients with severe diseases such as prostate, breast, and pancreatic cancer. Hao et al. presented a graphene-based, integrated portable nanosensing device for the detection of cytokine biomarkers in saliva. The biosensor employs an aptameric graphene-based field-effect transistor with HfO_2_ as a dielectric layer and an integrated processing circuit for the detection of cytokine concentrations. The biosensor reports a LOD of 12 pM (Figure 2c) [19]. H1N1, otherwise known as swine flu, is a virus that caused a worldwide outbreak starting in April 2009. The virus is easily spreading, and the World Health Organization declared a global outbreak raising the pandemic alert level to Phase 5 by 30th April 2009. Fast early detection is crucial to prevent further outbreaks in the future [27]. Ferguson et al. developed a magnetic integrated microfluidic electrochemical detector for quantification of H1N1 from throat swab samples. Integrating a multifunctional sample preparation chamber enables the testing of unprocessed samples into the biosensor without the need for external preparation or reagents. To demonstrate the device’s ability, they used H1N1 detection, which had a LOD of 10 TCID_50_ where TCID_50_ (median tissue culture infectious dose) [20].

Halitosis is a prevalent issue for most of the adult population. It is defined as an unpleasant odor emitted from the mouth, which may be caused by oral conditions including periodontal disease, chronic sinusitis and bronchiectasis [28]. Aliphatic polyamines in the oral cavity have been associated with halitosis and can occur from the breakdown of proteins and peptides or the degradation of amino acids. Piermarini et al. proposed a Prussian Blue screen-printed electrode electrochemical biosensor for the detection of biogenic amines in saliva. The sensor uses the hydrogen peroxide produced from the reaction with the diamine oxidase enzyme. The biosensor, coupled with portable instrumentation, showed a working range of 0.02 mM to 0.3 mM and a LOD of 0.01 mM [21]. Cortisol is a hormone used for regulation of blood pressure, cardiovascular function, and other metabolic activities. Cortisol levels have become a useful measurement for overall stress levels and disease levels in patients. Stevens et al. created a cortisol detecting biosensor utilizing a surface plasmon resonance system. For detection, cortisol specific monoclonal antibodies were used to develop a competition assay with a six-channel portable device. The biosensor reports a dynamic range of 1.5–10 ng/mL and a LOD of 1.0 ng/mL [22]. Platelet-derived growth factor BB (PDGF-BB) plays a role in regulating cell growth and division. It is necessary for increased healing with fibroblast activation and granulation tissue formation in the treatment of chronic dermal wounds. Ma et al. reported a biosensor for the detection of platelet-derived growth factor-BB. It employs a personal glucose meter which has the primary aptamer of PDGF-BB bound to the surface of streptavidin magnespheres paramagnetic particles from Promega Corporation (Madison, WI, USA). The streptavidin magnespheres react with an invertase-functionalized secondary aptamer of PDGF-BB to form a stable complex which results in the attachment of invertase on the paramagnetic particles. The invertase catalyzes the hydrolysis of sucrose to produce glucose for reading with the personal glucose meter. The biosensor reports a dynamic range of 1.0 × 10^−14^–3.16 × 10^−12^ M and a LOD of 2.9 fM [23]. Measurement of saliva conductivity can be used for the detection of dehydration. Dehydration is often associated with abnormalities in electrolyte balance allowing for a detection hydration status through the osmolality of a fluid. Hematological osmolality is a common index of hydration status. Dehydration and heat stress can lead to persistent damage to the kidneys which can lead to chronic kidney disease. Lu et al. developed a biosensor for the measurement of saliva osmolality via measurement of the conductivity of saliva samples. The sensor reports a sensitivity of 93.3% and a specificity of 80% [24]. Monitoring glucose in infants for diabetes treatment is especially challenging. García-Carmona et al. demonstrated a pacifier glucose biosensor to overcome some of the challenges of infant metabolite monitoring capable of detecting glucose in saliva. The biosensor is comprised of a Glucose-oxidase based enzyme detection electrode biosensor. It used glucose testing in adults to demonstrate the feasibility of the biosensor. They reported a LOD of 0.04 mM and a dynamic range of 0.1 to 1.4 mM. Figure 2d shows a figure of the pacifier biosensor [25].

### 2.2. Sweat

**Figure 3:**
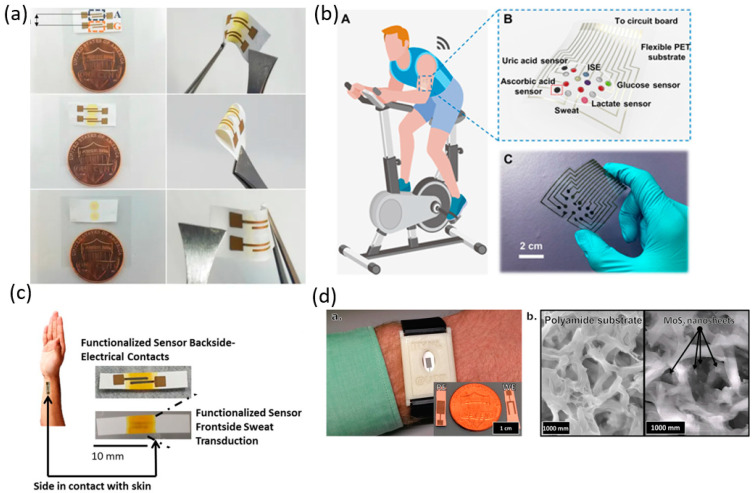
Portable biosensors for sweat biomarker detection. (**a**) Sweat sensor array showing fluid confinement in the active sensing region, sensor flexibility, and size comparison with one cent [29]. (**b**) Schematic illustration of a wearable sweat analysis patch mounted on human skin with a photograph of the actual patch [30]. (**c**) Wearable diagnostic sweat based biosensing and relative size of the developed sensor with RTIL and immunoassay functionalized semiconducting ZnO films on nanoporous polyamide substrates. The second part of the image shows the wicking of fluid in the active region of the sensor along with a schematic showing capture probe–target biomarker interaction in RTIL and immunoassay with ZnO thin film on a porous membrane within the wicked region of the fluid [31]. (**d**) Prototype and MoS_2_ nanosheet on polyamide membrane [32].

Sweat is another useful source of biomarkers for portable biosensors. Sweat sensors are often targeted towards wearable applications and allow for the noninvasive collection of samples. Human sweat is approximately 99% water with sodium chloride [12], but contains various health-related biomarkers including ascorbic acid, uric acid, metabolites like glucose and lactate, and electrolytes such as Na^+^ and K^+^. It is an attractive, less-painful alternative to blood samples for assessing a patient’s health. In the following section, sweat biomarkers for use with portable biosensors are summarized (Table 2). In addition, we show the recent examples of portable sweat biosensor along with the limits and dynamic ranges of detection (Figure 3).

Alcohol levels can be measured in sweat and show treatment response for conditions such as diabetes where alcohol consumption results in hypoglycemia. Additionally, alcohol is an important marker for the monitoring of drivers or individuals in safety-related work. Gamella et al. developed a biosensor based on a bienzyme amperometric composites sensitive to variation in ethanol concentration. The design enabled a dynamic range of 0.0005–0.6 g/L and a LOD of 0.0005 g/L [33]. Glucose is an important biomarker for monitoring and treating diabetes. Since alcohol and glucose are both relevant to diabetes monitoring and control, Bhide et al. presented a zinc oxide thin film integrated into a nanoporous flexible electrode biosensor to detect both glucose and alcohol. The biosensor can use low volumes of sweat for testing and both analytes have a dynamic range of 0.01–200 mg/dL with a LOD of 0.1 mg/dL. Figure 3a shows an image of the flexible biosensor [29]. Gao et al. reported a flexible, wearable biosensor capable of measuring both lactate and glucose. The sensor was used to test levels in sweat based on glucose oxidase, or lactate oxidase depending on the analyte of interest, immobilized within a permeable film. The sensor reported a LOD of 2.35 nA/µM for glucose [34]. Human sweat also contains useful ions that are often used to monitor a patient’s physiological state. Sodium ions in sweat can be used to diagnose diseases such as cystic fibrosis and autonomic and peripheral neuropathy where sweat regulation is affected. He et al. developed a multiplex biosensor for the detection of ions and electrolytes (Na^+^ and K^+^) as well as ascorbic acid (AA), uric acid (UA), glucose and lactate (Figure 3b). The sensor reports a LOD of 5 µM, 0.5 mM, 0.1 µM, 1 µM, 1 mM, 0.5 mM for Glucose, Lactate, UA, AA, Na^+^, K^+^ respectively. The dynamic range is reported as 25–300 μM, 5–35 mM, 2.5–115 μM, 20–300 μM, 5–100 mM, and 1.25–40 mM for Glucose, Lactate, UA, AA, Na^+^, K^+^ respectively [30]. Interleukin-6 is a pluripotent cytokine secreted by lymphoid and non-lymphoid cells. It has the potential for monitoring immune response during the treatment of cancer and is known to increase glucose intake along with influencing insulin activity. Munje et al. developed a biosensor comprised of room temperature ionic liquids with antibodies functionalized sensors on nanoporous, flexible polymer membranes (Figure 3c). They used the sensor to detect interleukin with a LOD of 0.2 pg/mL and 2 pg/mL for 0–24 h and 24–48 h post antibody sensor functionalization, respectively [31]. The chemiluminescence smartphone-based biosensor seen in the saliva section can also be used to monitor lactate can be found in sweat. The LOD for Lactate analysis in sweat is reported as 0.1 mmol/L [15]. Cortisol is another biomarker found in sweat. A cortisol detecting biosensor was designed with functionalized cortisol antibodies on MoS_2_ nanosheets integrated into a nanoporous flexible electrode (Figure 3d). The biosensor was targeting for a cortisol range of 8.16–141.7 ng/mL corresponding to a relevant cortisol level in human perspiration. The biosensor reports a dynamic range of 1–500 ng/mL and a LOD of 1 ng/mL [32].

### 2.3. Urine

**Figure 4:**
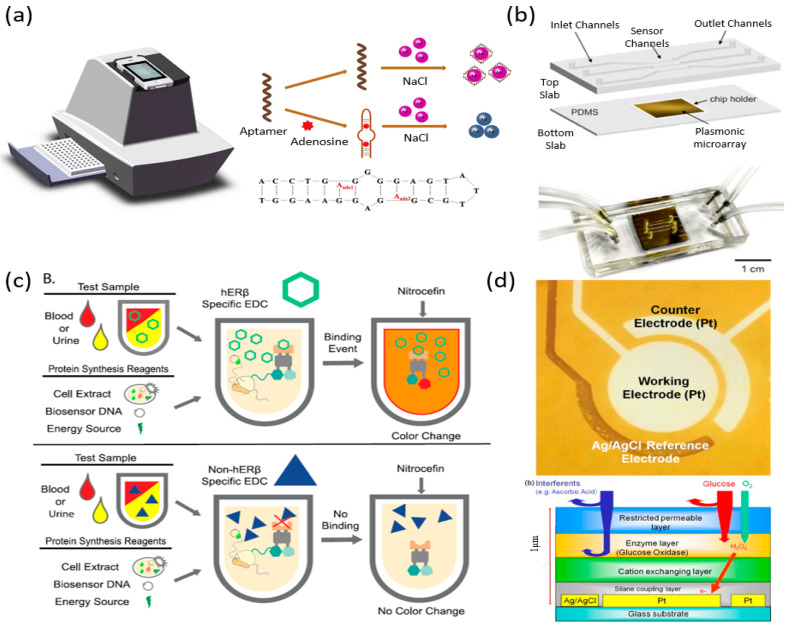
Portable biosensors for urine Biomarker biosensors detection. (**a**) The schematic of the bionic electronic-eye (E-eye) and the sensing mechanism of the colorimetric aptasensor for adenosine detection [35]. (**b**) Schematic and image of Soler’s Chlamydia trachomatis (CT) and Neisseria gonorrhoeae (NG) biosensor integrated with a microfluidic system [36]. (**c**) The RAPID biosensor assay. The presence of estrogen hERβ-specific ligands in the sample triggers a color change in the assay, which can be observed visually or more accurately measured using a spectrometer [38]. (**d**) Glucose sensor construction showing the Electrodes layout in H_2_O_2_ sensor and a Cross-sectional schematic of the sensor [39].

Urine is 95% water with sodium, phosphate, sulfate, urea, creatinine, proteins and kidney/liver byproducts which include metabolites and drugs. It has advantages in that it can be easily obtained in large quantities and is noninvasive [12]. Additionally, the lower concentration of protein, lipids and other high molecular weight compounds allows for less complex preparation. In this section, several analytes in urine are discussed (Table 3) with the recent development of portable biosensors (Figure 4).

Adenosine is a potential biomarker for detecting and monitoring lung cancer that can be found in urine. Zhou et al. developed a colorimetric aptasensor with a homemade biomimetic electronic eye for use in portable detection. The biosensor is capable of a dynamic range of 5.0 μM–60.0 μM and a LOD of 0.17 μM. A schematic of the adenosine biosensor and its operating principle can be seen in Figure 4a [35]. Chlamydia trachomatis (CT) and Neisseria gonorrhoeae (NG) are both sexually transmitted infection which is the first and second most reported bacterial infections. Soler et al. developed a portable biosensor for the detection of CT and NG in urine. It is comprised of an optically transparent gold nanohole sensor array functionalized with antibodies and a microfluidic system as shown in Figure 4b. The biosensor reports a LOD of 300 CFU/mL and 150 CFU/mL for CT and NG, respectively [36]. Neopterin is a useful biomarker as it provides information on the cellular immunity activation associated with oxidative stress. A biosensor designed by imprinting neopterin onto poly(ethylene- *co* -vinyl alcohol) as template molecules was developed by Huang et al. After imprinting, the template is removed, and the membrane can be used as a sensing element for electrochemical analysis of urine. The LOD for the biosensor was reported to be as low as 0.025 pg/mL [37]. Endocrine-disrupting chemicals such as diethylstilbestrol and bisphenol can disrupt naturally occurring endocrine control causing diseases and disorders such as cancer and epigenetic dysfunction. Salehi developed a portable biosensor (seen in Figure 4c) for the detection of chemicals that interact with estrogen receptor β (hERβ). The sensor consists of an allosterically activated fusion protein that contains the ligand-binding domain of a nuclear hormone receptor and (hERβ) is synthesized in cell-free protein synthesis. In previous work, they used β-lactamase instead of hERβ which shows the ability to change the sensor for many different endocrine disruptors. The dynamic range is reported to be 4–100 nM with a LOD of 4nM for urine [38]. Miyashita reported a urine glucose meter that uses immobilized glucose oxidase to detect glucose amperometrically. As discussed in previous sections, glucose levels are crucial to monitoring diabetes. The sensor can give results in six seconds and has a dynamic range of 0–2000 mg/dL. An image of the urine glucose meter and a diagram of its construction can be seen in Figure 4d [39]. Performance-enhancing drugs continue to be a problem in professional sports. Both dopamine and ephedrine can be detected in the urine of an individual who has used a performance-enhancing drug. Nikolelis developed a portable biosensor capable of detection of doping materials in the urine. The sensor is designed with a stabilized lipid membrane with an artificial receptor added before polymerization. The lipid film is then formed on microporous filters by polymerization of UV irradiation. The biosensor is capable of a dynamic range of 0 to 100 nM and a LOD of 10^−9^ M for both ephedrine and dopamine [40].

### 2.4. Blood

**Figure 5:**
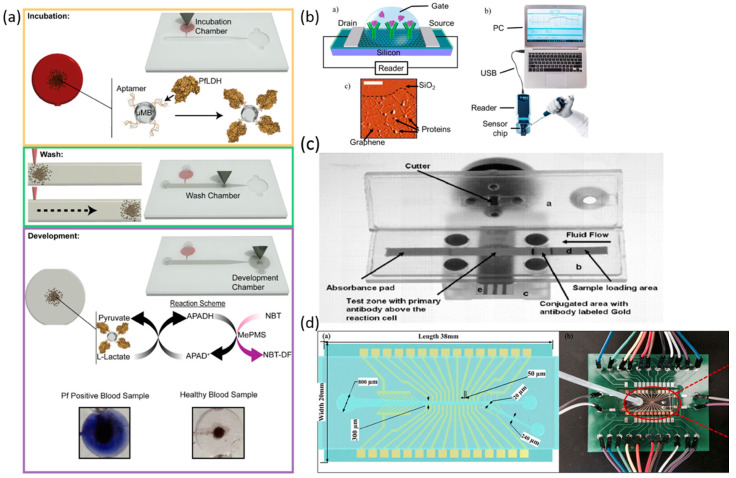
Portable biosensors for blood biomarker detection. (**a**) Operation and detection stages of the microfluidic APTEC biosensor [42]. (**b**) Diagram of the sensor element of the graphene biosensor chip with an AFM image of the graphene after successful protein attachment. In addition, an illustration of the entire sensor chip system [43]. (**c**) The schematic diagram of an IEB [47]. (**d**) Design sketch of the microfluidic chip; Photograph of the microfluidic chip and the peripheral control line design [49].

Blood has been the most widely used sample type due to a large number of disease-associated biomarkers and its less invasive collection procedure. Thus, it becomes a good source of biomarkers for portable biosensors. Blood sampling can be as simple as a finger prick with a small drop of blood used in a portable glucometer or full blood sampling which requires a trained individual to draw. Blood, plasma, and serum testing show a good correlation between pharmacologic effect and compound concentration. Blood cells are suspended in plasma and water and it contains many proteins, glucose, mineral ions, hormones, carbon dioxide and platelets [12]. In this section, we summarize many types of blood biomarkers for portable biosensors (Table 4). In addition, we describe the recent development of portable blood biosensors along with their limits and ranges of detection (Figure 5).

The effort to eradicate malaria has been able to decrease mortality by 48% from 2000 to 2015, but it still remains endemic in 97 countries. An aptamer-tethered enzyme capture assay for POC diagnosis of Plasmodium falciparum was developed by Dirkzwager et al. [41]. The sensor captures Plasmodium falciparum lactate dehydrogenase from samples and uses its enzymatic activity to generate a blue color in response. The sensor utilizes only small sample volumes of 20 µL and has a LOD in the ng/mL range. Fraser et al. present the portable biosensor (Figure 5a) by coating aptamers onto magnetic microbeads for magnet-guided capture. They employ three separate microfluidic chambers for the detection of Plasmodium falciparum lactate dehydrogenase enzyme. The LOD is reported to be 250 parasites/µL [42]. Zika is a vector-borne viral infection that can cause brain defects in fetuses. Afsahi et al. developed a biosensor for Zika detection by covalently linking graphene with monoclonal antibodies for the detection of Zika antigens. A Diagram of the sensor can be seen in Figure 5b. The biosensor responds to Zika antigens with a change in capacitance, allowing for the detection of the virus in samples. The LOD is reported to be 0.45 nM [43]. Arboviral disease, dengue hemorrhagic fever, or dengue shock syndrome can all be caused by the dengue virus. Zaytseva developed a biosensor built of generic and specific serotype-specific DNA probes. Through the use of a reflectometer, liposome immobilized in capture zones can be quantified, which directly correlates to viral RNA. The LOD is reported to be 50 RNA molecules for serotype 2, 500 RNA molecules for serotypes 3 and 4, and 50,000 molecules for serotype 1 [44]. Yersinia Pestis is the etiological agent of plague and has resulted in three pandemics. A Biosensor for detection of antibodies of Yersinia Pestis was created using a sandwich immunoassay with immobilized Escherichia coli on the optic fiber probes labeled with Cy-5 as a detection antigen. The *Yersinia Pestis* biosensor reports a LOD of 10 ng/mL with 100% sensitivity and 94.7% specificity in rabbit serum [45].

Exposure to organophosphorus insecticides has recently been found to inhibit the enzyme activity of acetylcholinesterase in the central and peripheral nervous systems. Trichloropyridino is the primary metabolite marker when exposure to organophosphorus insecticides occurs. Zou et al. recently developed a portable biosensor for Trichloropyridinol sensing. The biosensor contains an immunochromatographic test strip assay with a quantum dot label integrated into a portable fluorescent sensor. The biosensor exhibited a dynamic range of 1–50 ng/mL and a LOD of 1.0 ng/mL [46]. Wang et al. presented a new immunochromatographic electrochemical biosensor (IEB) for the detection of trichloropyridinal (TCP) in blood seen in Figure 5c. The sensor utilizes an immunochromatographic test strip for a competitive immunoreaction along with a disposable screen-printed carbon electrode for analysis of captured horseradish peroxidase labeling. The biosensor exhibited a dynamic range of 0.1–100 ng/mL with a LOD of 0.1 ng/m [47]. Copper ions (Cu^2+^) are essential to human life, but at high concentrations, they can cause adverse health effects. A biosensor developed by Ming et al. using Cu^2+^-dependent DNA ligation DNAzyme enabled Copper ion detection in a glucose meter. The biosensor reportedly has a dynamic range of 10–600 mg/dL and has a possible LOD of 1 nM [48]. The estrogenic endocrine disruptor biosensor previously described in the urine section can also be used for the detection of EED’s in blood. The dynamic range is reported to be 8–300 nM with a LOD of 8 nM for blood [38].

Hemolytic anemia is a disorder in which red blood cells die faster than they can be made, causing the concentration of dead red blood cells to be much higher when compared to a healthy individual. Sang et al. developed a biosensor consisting of a multifunctional dielectrophoresis manipulation device and a surface stress biosensor to separate and detect red blood cells for the detection of hemolytic anemia. The biosensor allows for the detection of live and dead red blood cells, and the diagnosis of Hemolytic anemia can be made from capacitance reading from the biosensor. The device was successfully able to sort live/dead red blood cells. A figure of the biosensor can be seen in Figure 5d. Future tests will be needed to determine how the capacitance measurement can be used to calibrate with the severity of the disease [49]. Methotrexate is a commonly used drug in the treatment of cancers and some autoimmune diseases due to its immunosuppressive properties. Zhou et al. developed a biosensor sensor comprised of a carbon-based composite fixed to an electrode surface supporting redox cycling. The dynamic range was reported to be 0.01–45 µM with a LOD of 45 nM [50].

### 2.5. Tears/Breath

**Figure 6:**
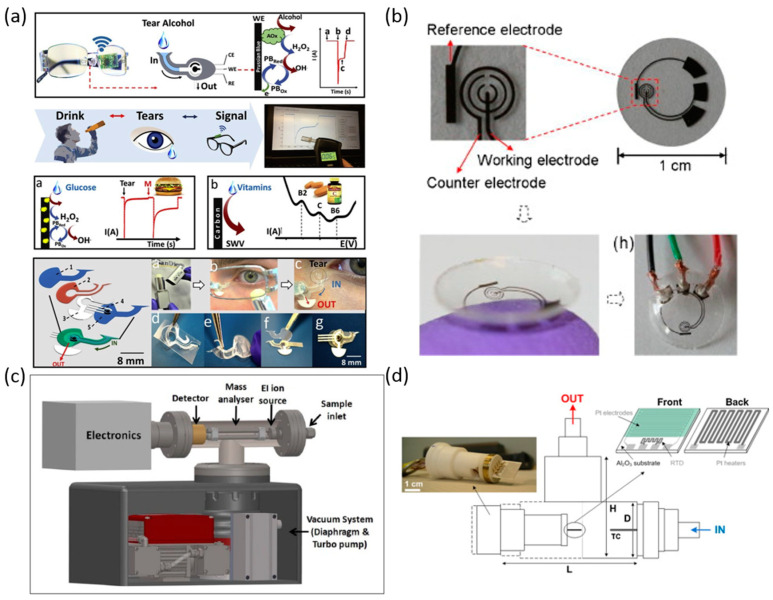
Portable biosensors for tears/breath biomarker detection. (**a**) Eyeglasses-based Fluidic Device [52]. (**b**) Prototype contact biosensor for glucose sensing [53]. (**c**) Schematic diagram of the portable QMS and vacuum system [55]. (**d**) Schematic and image of the portable breath acetone monitor with Si:WO3 gas sensors [56].

In this section, we discuss portable biosensors that use tears and breath as the specimen. Tears can provide an easily collectible convenient means for monitoring and testing biomarkers. For biosensors that passively collect tears from the surface of the eye, the collection is no more invasive than wearing a contact lens. However, if larger quantities are required, portable sampling could prove to be difficult. Human breath also contains thousands of potential disease and chemical exposure biomarkers [51]. Using breath as a specimen is very portable and noninvasive to collect. A trained individual would not be necessary for the collection. Depending on the sample volume required, collection can be as natural as breathing into a bag or through a chamber and could be repeated as often as needed. Here, we summarize many types of biomarkers in tears and breath for portable biosensors (Table 5). In addition, we describe the recent development of portable biosensors along with their limits and ranges of detection (Figure 6).

Sempionatto et al. developed a portable biosensor for the detection of alcohol, glucose, and vitamins B2, B6 and C in tear samples seen in Figure 6a. The biosensor consisted of an electrochemical alcohol-oxidase biosensor integrated with a microfluidic system. The sensor-enabled noninvasive, real-time testing but did not include a dynamic range or LOD in the report [52]. Yao et al. reported a contact lens integrated with an amperometric glucose sensor capable of the detection of glucose molecules from tear samples seen in Figure 6b. The sensor consisted of microstructures on a polymer substrate shaped into a contact lens. The sensor maintained a linear response to a range of 0.1–0.6 mM and has a LOD of 0.01 mM [53]. Chu et al. also demonstrated glucose monitoring with a contact lens which consists of an enzyme immobilized electrode on the surface of a PDMS lens. The system reported having a dynamic detection range of 0.03 to 5 mmol/L [54]. *Helicobacter pylori* can be detected in breath samples, which has been correlated to chronic gastritis, gastric and duodenal ulcers, and gastric cancer. Sreekumar et al. developed a noninvasive biosensor (Figure 6c) capable of detection of helicobacter pylori. The biosensor consisted of a portable quadrupole mass spectrometer (QMS) system measuring carbon-13 levels in breath samples. The results from clinical samples showed an overall agreement of 87% when compared to the laboratory isotope ratio mass spectrometry (IRMS) results [55]. Acetone in breath samples appears in a relatively high concentration of approximately 100–500 ppb. Acetone content in breath can be used as supplemental information for Diabetes treatment. Righettoni et al. fabricated a biosensor (Figure 6d) consisting of Si-doped epsilon-WO3 nanostructured films. The acetone sensor demonstrated to have a LOD of 20 ppb [56]. Exhaled breath chemicals contain both volatile and non-volatile compounds which can be indicative of different diseases. A biosensor consisting of a combination of a differential mobility spectrometer with a gas chromatograph and an electrospray ionization module is examined. The biosensor is capable of detecting aa large amount of chemical analytes but reports values of a LOD for toluene (200 ppb) and angiotensin (1 pM) [51]. Asthma, chronic obstructive pulmonary disease, pneumonia, and sleep apnea are all potentially cause respiratory acidosis. Respiratory acidosis is a condition where the lungs are unable to remove enough carbon dioxide produced by the body. Bagchi et al. reported a biosensor for the detection of CO_2_ in breath by immobilizing carbonic anhydrase enzyme on an electrode assembly. The device reports a linear electrochemical response to CO_2_ from 160–2677 ppm and a LOD of 0.132mV/ppm [57].

## 3. Discussion

Some of the common analytes have been repeated throughout the article (Table 6). This goes to show that not only can analytes be found in different biological samples, but that the LOD can vary widely between biosensors based on design and implementation. The commonly repeated analytes are lactate, glucose, and alcohol. Both glucose and lactate can be used for multipurpose monitoring, which can further explain why they are commonly being researched through many different biological samples. Alcohol sensing can also be used for multiple purposes, such as in the treatment of diabetes, but can be used for monitoring drivers or individuals in safety work.

**Figure 7:**
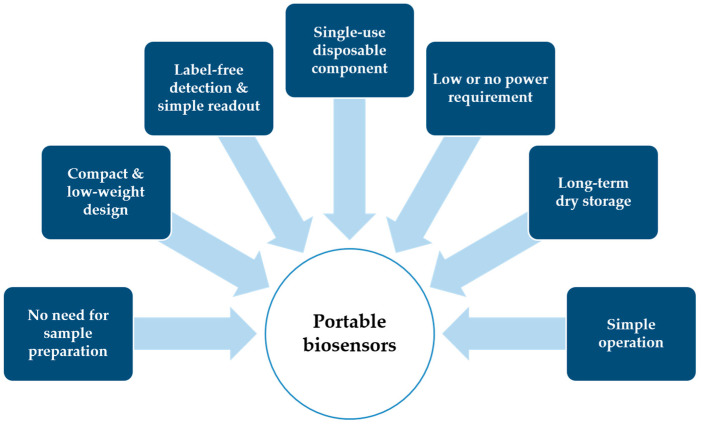
Key requirements for portable biosensors.

As seen in the biosensors and their target biomarkers, many portable devices have been developed for pathogen detection and health monitoring. Still, the portability of the systems has not been discussed in much detail. While some portable biosensors could be moved by vehicle and set up in remote locations, others may need to be able to be worn or handheld. The portability of biosensors for on-site diagnosis can be limited due to various issues, including sample preparation techniques, fluid-handling techniques, the limited lifetime of biological reagents, device packaging, integrating electronics for data collection/analysis, and the requirement of external accessories and power. This limits the ability of researchers and physicians to identify portable biosensors, which can be applied to fields and practices. The well-defined portability for biosensors could provide users a better idea of how mobile and compact they are for field use. Here, we summarize the portability requirements for the classification categories of the biosensors in Figure 7.

Note that all recommended requirements are not currently quantifiable and would need to be further studied and classified to create portability classification requirements. The above list also may not contain all pertinent information to the portability of a biosensor but provides recommendations for starting components to consider.

In the section of the analyte, we have seen sample preparation techniques ranging from centrifuging to chemical addition to an analyte. Biological samples contain many different proteins, lipids, and contaminants that can affect how samples need to be prepared and, ultimately, the outcome of tests. Often the analytes of interest are in a much lower concentration when compared to other substances that make up the remainder of the sample [12]. Sample preparation can be one of the limiting factors causing a biosensor system to be portable. While the biosensor itself meets the portability requirements, if the sample preparation requires non-portable laboratory equipment, the testing, diagnosis, and monitoring cannot be made remotely. Strand et al. gives an example of biosensing issues from existing sample preparation techniques. Metabolites can be found in human breath and are known to be up or down-regulated in various disease states. Measuring metabolites in exhaled breath can be used for the diagnosis or treatment of diseases, including cancer, asthma, and respiratory infections. However, analysis is currently limited due to the inability to concentrate the analytes of interest before testing. Strand developed a chip-based polymeric pre-concentration device with the ability to absorb and desorb breath volatiles for analysis [58].

**Figure 8:**
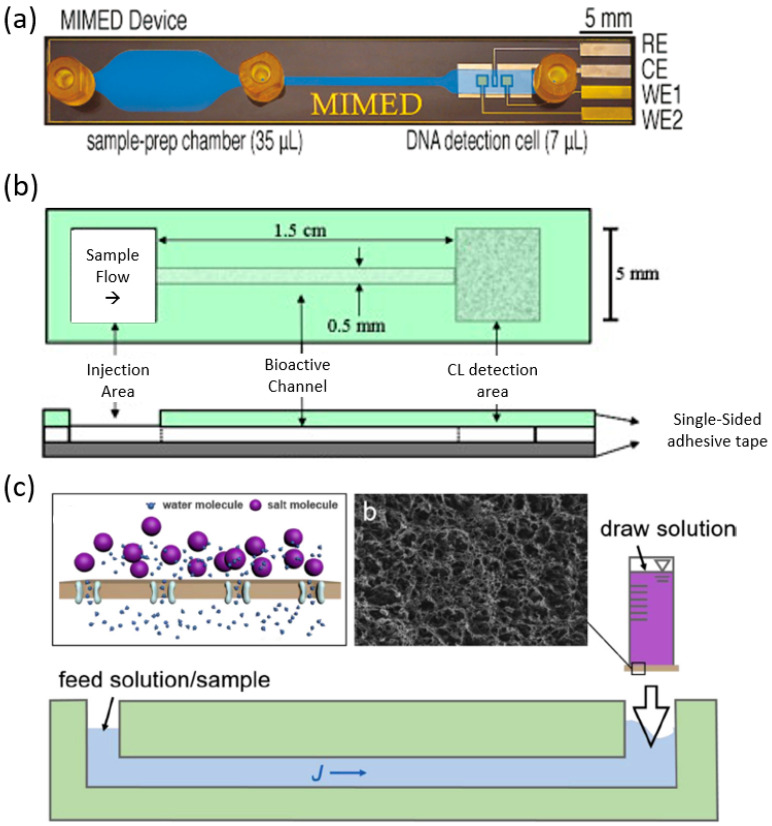
Microfluidic integrated biosensors. (**a**) Magnetic Integrated Microfluidic Electrochemical Detector device for detection of H1N1 [20]. (**b**) Schematic of a paper microfluidic biosensor for uric acid determination [59]. (**c**) Schematic of a microfluidic device driven by osmotic pressure [60].

One possibility to integrate portable sample preparation into a portable biosensor is to integrate microfluidics into the system. Microfluidic integrated biosensors have great potential to help make biosensors more portable. The microfluidic chip can be tiny and encompass an entire sample preparation method that may otherwise require a laboratory. Ferguson et al. demonstrate an integrated microfluidic system, which enables sequence-specific viral RNA-based pathogen detection without the need for pre-processing of throat swap samples seen in Figure 8a. [20]. Yu et al. proposed a paper-based microfluidic biosensor for uric acid determination, which gives the advantage of working with small volumes of reagent and having short reaction times Figure 8b [59]. Some microfluidic devices require external pumps or large power supply to operate, limiting the portability of the system. Chuang et al. show a microfluidic device utilizing capillary action negating the need for an external pump, further increasing the portability of sample preparation seen in Figure 8c [60].

## 4. Conclusions and Outlooks

Despite the recent progress of portable biosensors, many challenges still remain before the implementation of portable biosensors in practical applications. The following requirements need to be considered for future development. First, the sample collection and preparation method is simple, easy to use, and does not involve costly and time-consuming processes. In that sense, current lab-on-a-chip technologies, such as microfluidics, can provide a solution for this goal. Also, sample types such as saliva, sweat, urine, and breath offer an excellent opportunity for noninvasive sample collection. Second, the sensitivity and specificity improvement is an ever-lasting goal in biosensor development. This might be one of the major barriers to the application of portable biosensors. While many biosensors reported in the literature perform well in laboratory settings, they may encounter a series of problems in-field use with real samples. Thus, it is essential to develop highly sensitive and specific portable biosensors whose performance is comparable to conventional laboratory testing. Third, cost-effective packaging and manufacturing methods should be accompanied by the fabrication of portable biosensors. Microfabrication technology is widely used for manufacturing, but it needs expensive production equipment. New manufacturing technologies, such as 3D-printing, are capable of reducing fabrication costs and enabling mass production. We believe that these challenges will be gradually solved in the future, leading to successful portable biosensors, which allow us to monitor our health status at any time and place.

In conclusion, the wide variety of analytes and the associated portable biosensors provides an excellent opportunity for POC diagnostics as well as remote health care monitoring. While biosensors can be made portable, the associated sample collection and preparation play a crucial role in developing entirely portable systems that can truly expand the possibilities of diagnostics, especially in areas of limited resources and for immediate, on-demand measurement. Sample types, such as saliva, sweat, urine, and breath, offer an excellent opportunity for noninvasive sample collection. While blood provides a wide range of analytes for testing, its collection can be more invasive. The processes described in the analyte section of this paper can often be altered or expanded upon for use with different analytes to provide the ability for multiplexing biomarkers with just a single sensor. Lastly, the portability of biosensors needs to be classified. Portable is a vague statement that can cause confusion and errors in the developing field. Without classification, the practical use and viability of biosensors, sample collection, and preparation cannot be fully applied to on-site diagnostics.

## Figures and Tables

**Table 1:** Summary of Portable Biosensors for Salivary Biomarker Detection.

Biomarker	Target Disease/Area	Sensor Type	Detection Limit * Sensitivity ** Specificity	Dynamic Range	Analysis Time
Lactate [14]	Respiratory insufficiency, shocks, heart failure and metabolic disorders	Modified screen printed electrode	0.01 mM	0.025–0.25 mM	<60 s
Lactate [15]	Diabetes, sports medicine, critical care	3D printed chemiluminescence biosensor	0.1 mmol/L	NA	<5 min
Lactate [16]	Clinical diagnosis, sport physiology and food analysis	Cloth-based electrochemiluminescence (ECL)	0.035 mM	0.05–2.5 mM	NA
Streptococcus [17]	Streptococcus Pyogenes	Impedimetric Immunosensor	NA	100 to 10^5^ cells/10 µL cumulative incubation 100 to 10^4^ cells/10 µL single-shot	NA
Avian Influenza Virus [18]	Avian influenza	Impedance biosensor	1 × 10^2.2^ ELD_50_/mL Tracheal = 100% * Cloacal = 55% *	NA	<1 h
Cytokine biomarkers [19]	Disease detection such as cancer	Graphene-based fully integrated portable nanosensing biosensor	12 pM	NA	Real-time
H1N1 [20]	Influenza detection	Magnetic Integrated Microfluidic Electrochemical Detector	10 TCID50	NA	3.5 h
Biogenic Amines [21]	Halitosis	Diamine Oxidase Electrochemical screen printed electrode Biosensor	1 × 10^−5^ M	2 × 10^−5^–3× 10^−4^ M	NA
Cortisol [22]	Stress	Surface plasmon resonance biosensor	1.0 ng/mL	1.5 ng/mL–10 ng/mL	<10 min
PDGF [23]	Cell growth and division	Aptamer-based biosensor PGM	2.9 fM	1.0 × 10^−14^ M to 3.16 × 10^−12^ M	20 min
Saliva Conductivity [24]	Dehydration and Kidney function	Au Electrode biosensor	93.3% * 80% **	NA	Real-time
Metabolites (Glucose) [25]	Metabolite pacifier biosensor for infants	Glucose-oxidase based enzyme detection electrode biosensor	0.04 mM	0.1 to 1.4 mM	Real-time

**Table 2:** Summary of Portable Biosensors for Sweat Biomarker Detection.

Biomarker	Target Disease/Area	Sensor Type	Detection Limit * Sensitivity	Dynamic Range	Analysis Time
Alcohol [33]	Noninvasive measurement	Bienzyme amperometric composite biosensors	0.0005 g/L	0.0005–0.6 g/L	Real-time
Glucose [29]	Diabetes	Zinc Oxide Thin film nanoporous electrode biosensor	0.1 mg/dL	0.01–200 mg/dL	NA
Multiplexed (Metabolites/electrolytes/temperature) [34]	Physiological monitoring	Flexible sweat sensor array with wireless FPCB	2.35 nA/μM Glucose * 220 nA/mM Lactate *		NA
glucose, lactate, ascorbic acid, uric acid, Na^+^ and K^+^ [30]	Multipurpose healthcare monitoring	Silk fabric–derived intrinsically nitrogen (N)–doped carbon textile (SilkNCT) flexible biosensor	Glucose: 5 μM Lactate: 0.5 mM UA: 0.1 μM AA: 1 μM Na^+^: 1 mM K^+^: 0.5 mM	Glucose: 25 to 300 μM Lactate: 5 to 35 mM UA: 2.5 to 115 μM AA: 20 to 300 μM Na^+^: 5 to 100 mM K^+^: 1.25 to 40 mM	Real-time
Interleukin [31]	Immune response	BMIM[BF4] RTIL stability enhancing capture probe immunoassay functionalized ZnO thin films deposited on nanoporous polyamide membrane biosensor	0.2 pg/mL for 0–24 h and 2 pg/mL for 24–48 h post-antibody sensor functionalization	0.2–200 pg/mL continuous detection	NA
Lactate [15]	Diabetes, sports medicine, critical care	3D printed chemiluminescence biosensor	0.1 mmol/L	NA	<5 min
Cortisol [32]	Stress	Non-faradaic label-free cortisol biosensor	1 ng/mL	1–500 ng/mL	Continuous for 3+ hours

**Table 3:** Summary of Portable Biosensors for Urine Biomarker Detection.

Biomarker	Target Disease/Area	Sensor Type	Detection Limit * Sensitivity	Dynamic Range	Analysis Time
Adenosine [35]	Lung Cancer	Colorimetric aptasensor	0.17 μM	5.0 μM–60.0 μM	<20 min
Chlamydia trachomatis [36]	Chlamydia	Nanoplasmonic biosensor	300 CFU/mL	NA	Real time
Neisseria gonorrhoeae [36]	Gonorrhoeae	Nanoplasmonic biosensor	150 CFU/mL	NA	Real time
Neopterin [37]	Aging	Molecularly Imprinted Polymer integrated Potentiostat	0.025 pg/mL 0.041 pg/mL compared to reference of 35–55 ng/mL *	NA	NA
Estrogenic Endocrine Disruptor [38]	Obesity, birth defects, cancer, reproductive impairment	In-vitro Detection biosensor platform	urine 4 nM Blood 8 nM	4–100 nM, urine	2.5 h
Glucose [39]	Diabetes	Micro-Planer amperometric biosensor	NA	0–2000 mg/dL	6 s
Dopamine [40]	Doping	Stabilized lipid Membrane optical Biosensor	10^−9^ M	0 to 100 nM	<1 min
Ephedrin [40]	Doping	Stabilized lipid Membrane optical Biosensor	10^−9^ M	0 to 100 nM	<1 min

**Table 4:** Summary of Portable Biosensors for Blood Biomarker Detection.

Biomarker	Sample Type	Target Disease/Area	Sensor Type	Detection Limit * Sensitivity ** Specificity	Dynamic Range	Analysis Time
Malaria [41]	Whole blood	Malaria−	Aptamer Tethered Enzyme Capture assay	4.9 ng/mL	NA	<1 h
Malaria [42]	Blood	Malaria	Aptamer-Tethered Enzyme Capture (APTEC) biosensor	250 parasites/µL	NA	<20 min
Zika [43]	Simulated Serum	Zika	graphene-based biosensor	0.45 nM	NA	Real time
Dengue [44]	Blood	Dengue fever	multi-analyte biosensor based on nucleic acid hybridization and liposome signal amplification	50 RNA molecules for serotype 2, 500 RNA molecules for serotypes 3 and 4, and 50,000 molecules for serotype 1	NA	<25 min
Yersinia Pestis Antibody [45]	Rabbit serum	Etiological agent of plague	Antigen sandwich method using a portable fiber optic biosensor	10 ng/mL 100% * 94.7% **	NA	40 min
Trichloropyridino [46]	Rat Blood	Exposure to organophosphorus insecticides	Quantum Dot integrated Fluorescent biosensor	1.0 ng/mL	1–50 ng/ml	15 min
Trichloropyridino [47]	Rat plasma	Exposure to organophosphorus insecticides	Immunochromatographic electrochemical biosensor	0.1 ng/ml	0.1–100 ng/ml	15 min
Copper [48]	Serum	Copper Toxicity	Cu^2+^-dependent DNA ligation DNAzyme PGMs	1 nM possible	10–600 mg/dL	NA
Estrogenic Endocrine Disruptor [38]	Blood	Obesity, birth defects, cancer, reproductive impairment	In-vitro Detection biosensor platform	8 nM	8–300 nM in blood 4–100 nM urine	2.5 h
Red blood [49]	Blood	Anemia	Surface Stress Biosensor	NA	NA	NA
Anti-Cancer Drugs [50]	Blood	Toxicity	Novel Tungsten Phosphide Embedded Nitrogen-Doped Carbon Nanotubes biosensor	45 nM	0.01–45 µM	NA

**Table 5:** Summary of Portable Biosensors for Tears/Breath Biomarker Detection.

Biomarker	Sample Type	Target Disease/Area	Sensor Type	Detection Limit * Sensitivity	Dynamic Range	Analysis Time
Alcohol/Glucose/Vitamins (B2,B6,C) [52]	Tear	Various disease/Health Monitoring	Alcohol-oxidase (AOx) biosensing fluidic system	NA	NA	Real-time
Glucose [53]	Tear	Diabetes	Amperometric glucose biosensor	0.01 mM 240 uA/(mM·cm^2^) *	Linearity 0.1–0.6 mM	20 s
Glucose [54]	Tear	Diabetes	SCL-biosensor	NA	0.03–5.0 mmol/L	Real-time
helicobacter pylori [55]	breath	Chronic gastritis/(gastric/duodenal ulcers)/gastric cancer	Quadrupole mass Spectrometer biosensor	NA	NA	NA
Acetone [56]	Breath	Various disease/Health Monitoring	portable Si:WO3 gas sensors	20 ppb	NA	10–15 s
Volatile and non-volatile biomarkers [51]	Breath	Disease or chemical exposure	Differential Mobility Spectrometry	Toluene: 200 ppb Angiotensin: 1 pM	NA	Near real-time
CO_2_ [57]	Breath	Respiratory health	Carbonic Anhydrase-Based enzyme biosensor	0.132 mV/ppm *	160–2677 ppm CO_2_ linear response	12 s

**Table 6:** Summary of Commonly Repeated Analytes.

Biomarker	Sample Type	Target Disease/Area
Lactate	Saliva and sweat	Respiratory insufficiency, shocks, heart failure, metabolic disorders, diabetes, sports medicine, critical care, and food analysis
Glucose	Sweat, urine, and tears	Diabetes and general healthcare monitoring
Alcohol	Sweat tears, and breath	BAC for drivers and diabetes treatment for hypoglycemia prevention
